# Impact of methylphenidate formulation on treatment patterns and hospitalizations: a retrospective analysis

**DOI:** 10.1186/1744-859X-5-5

**Published:** 2006-04-10

**Authors:** Jason E Kemner, Maureen J Lage

**Affiliations:** 1 Associate Director Outcomes Research, McNeil Consumer and Specialty Pharmaceuticals, Fort Washington PA 19034, USA; 2 Managing Member, HealthMetrics Outcomes Research, Groton CT, USA

## Abstract

**Background:**

While stimulant therapy has been shown to be effective in the treatment of attention-deficit/hyperactivity disorder (ADHD), there is less information concerning differences between alternative stimulant medications. The purpose of this study is to examine how different formulations of methylphenidate (MPH) affect treatment patterns and hospitalizations.

**Methods:**

From a large claims database we retrospectively identified individuals age 6 or older who were diagnosed with ADHD and who received either once daily, extended-release oral system methylphenidate (OROS^® ^MPH) (e.g., Concerta^®^) or three-times daily immediate-release generic methylphenidate (TID MPH). There were 5,939 individuals included in the analysis – 4,785 who initiated therapy with OROS MPH and 1,154 who initiated therapy with TID MPH. We used Analyses of Covariance (ANCOVAs) to examine differences in treatment patterns between individuals who initiated therapy on OROS MPH and those who initiated therapy on TID MPH. We used logistic and negative binomial multivariate regressions to examine the probability of being hospitalized and the hospital length of stay.

**Results:**

Controlling for demographic characteristics, patient general health status, and comorbid diagnoses, significantly fewer individuals who initiated therapy with OROS MPH had a 15-day gap in therapy (85% vs. 97%, p < 0.0001 or a 30-day gap in therapy (77% vs. 95%, p < 0.0001) or switched to another ADHD medication (27% vs. 68%, p < 0.0001). Individuals who initiated therapy with OROS MPH stayed on therapy significantly longer (199 vs. 108 mean days, p < 0.0001) and more individuals received medication for 90% (24% vs. 5%, p < 0.0001), 80% (29% vs. 7%, p < 0.0001), or 75% (30% vs. 7%, p < 0.0001) of the days during the first year post initiation of therapy. Individuals who initiated therapy on OROS MPH were also significantly less likely to be hospitalized (odds ratio = 0.67, p = 0.0454) and stayed, on average, 0.69 fewer days in the hospital (p = 0.0035).

**Conclusion:**

Results demonstrate that among individuals diagnosed with ADHD who receive either OROS MPH or TID MPH, the use of OROS MPH is associated with fewer gaps in medication, less switches in medication, and more days on intent-to-treat therapy. In addition, use of OROS MPH compared to TID MPH was associated with improved outcomes, as measured by the reduced use of hospitalizations.

## Background

ADHD is one of the most frequently diagnosed childhood mental health conditions, with a prevalence of 8–10% in school age children[1]. Children diagnosed with ADHD can suffer from academic impairments, social dysfunction, and a higher risk of both cigarette smoking and substance abuse [2,3]. In addition, Rowe, Maughan, and Goodman (2004) found children or adolescents diagnosed with ADHD to be more likely to have unintentional injuries [4] , while other research has found young adults diagnosed with ADHD to be at increased risk for driving accidents [5-7].

Although ADHD is typically thought of as a childhood condition, it has been estimated that the condition persists into adulthood for 10–60% of individuals who were diagnosed as children [8,9]. As with the childhood population, there are significant costs associated with ADHD in the adult population. Specifically, adults with ADHD have been found to have larger medical costs [10] , less education [11] and higher rates of incarceration [12]. In addition, adults with ADHD are less likely to be employed [13,14] , while those employed are more likely to perform poorly, change employment, or quit their jobs [15,16].

Most commonly, stimulants are prescribed as first-line therapy for ADHD, with the American Academy of Pediatrics ADHD treatment guidelines stating that there is strong evidence for the use of stimulant medication [17]. While stimulant therapy has been shown to be effective in general [18,19] , the overall effectiveness of therapy also depends upon patient adherence. For example, Charach, Ickowicz, and Schachar (2004) examined adherence to stimulants over a 5 year period and found that, after five years, adherents showed greater improvement in teacher-reported symptoms than those off medications or those non-adherent to medication [18].

While stimulant therapy has been shown to be effective, there is less information concerning differences between the various stimulant medication formulations. The purpose of this research was to compare treatment patterns and outcomes of individuals who initiated therapy on differing stimulant medication formulations. Specifically, the analyses compared those who initiated therapy with TID MPH and those who initiated therapy with OROS MPH. At the outset, we hypothesized that the easier treatment regimen associated with once-daily OROS MPH would be associated with improved patient adherence and improved patient outcomes compared to TID dosing of immediate-release (IR) MPH. In this analysis, we measured patient outcomes by hospitalizations, a variable that contains implications for service planning and relates to injury rates.

## Methods

Data for this analysis came from the Integrated Health Care Information Services (IHCIS) National Managed Care Benchmark Database. This fully de-identified, HIPAA (Health Insurance Portability and Accountability Act)-compliant database includes complete medical histories for more than 17 million managed-care lives. Laboratory results, hospitalization data, pharmacy data, and complete mental health data are available from this database, in addition to patient demographics. The data undergoes a rigorous data quality review prior to its addition to the database and the data conforms to basic data validity norms. The data spanned the period February 1, 2000 to December 31, 2002.

Patients were eligible for inclusion in the analysis if they received a diagnosis of ADHD based upon International Classification of Diseases, Ninth Revision (ICD-9) codes of 314.00 or 314.01, and received either OROS MPH or TID MPH. Patients' records were indexed six months before and twelve months after first receiving the drug of interest. In order to focus on ADHD, we excluded individuals from the sample if they were diagnosed with bipolar disorder (ICD-9 296.4x – 296.8x), schizophrenia (295.xx), paranoia (297.xx), other psychotic disorders (290.xx-294.xx, 296.24, 296.34, 296.9x), Alzheimer's (331.0x), Parkinson's (332.xx), or mental retardation (317.xx – 319.xx). We also excluded individuals from the analysis if they received either OROS MPH or TID MPH in the six-month pre-period. In addition, for eligibility, individuals had to be at least age 6 and have continuous insurance coverage during the pre- and post-periods. There were 5,939 individuals who met the above criteria – 4,785 who received OROS MPH and 1,154 who received TID MPH.

The analysis focused on the differences in medication usage patterns and hospitalizations between individuals who initiated treatment with OROS MPH and those who initiated treatment with TID MPH. We examined several medication usage patterns, including gaps in therapy, switches in ADHD medication, number of days on therapy, and treatment adherence. We constructed two variables to capture gaps in therapy: *Gap15 *was defined as a 15-day or greater gap between the end of one ADHD medication prescription and the start of the next ADHD medication prescription or cessation of ADHD medication prior to 15 days before the end of the post-period of analysis; and *gap30 *was defined analogously to *gap15 *but with 30-day gaps in medication. We defined a *switch *as a cessation of treatment on the initial ADHD medication (either OROS MPH or TID MPH) and initiation of treatment with an alternative ADHD medication (either the other intent-to-treat medication or one of the following: twice-daily methylphenidate, Ritalin™, Ritalin LA™, Adderall™, Adderall XR™, or Metadate CD™), while we defined *switchitt *as cessation of treatment with OROS MPH and initiation of treatment with TID MPH, or vice versa. Finally, we defined adherence in terms of the number of days that the individual received the intent-to-treat (ITT) medication over the 365 day post-period, with *adherence90*, *adherence80*, and *adherence75 *receiving the ITT medication for 90%, 80%, and 75% of the post-period, respectively.

We used four classes of independent variables in this study: demographic characteristics, patient general health status, co-morbid diagnoses, and medication use. Patient demographic characteristics consisted of the individuals' age, sex, region, and type of insurance coverage. We proxied patient general health status as the number of distinct ICD-9 codes (based upon three digits) with which the individual was diagnosed during the six month pre-period. We based comorbidities upon an inpatient or outpatient diagnosis and classified them using ICD-9 codes. Previous research has found that individuals diagnosed with ADHD may be more likely to have comorbid alcohol or drug abuse (ICD-9 291.xx, 292.xx, 303.xx, 304.xx, or 305.xx), accidents or injuries (ICD-9 Exx.xx), and one or more of the following mental illness diagnoses: anxiety (ICD-9 300.00, 300.01, 300.02, 309.81, 300.2x or 300.3x), depression (ICD-9 of 296.2x, 206.3x, 300.4x, 309.0x, or 309.1x), or oppositional disorder (ICD-9 313.81) [5,10,20-24]. Finally, we hypothesized that differences in ADHD medication, specifically the use of once-daily OROS MPH compared to TID MPH, might affect both medication treatment patterns and outcomes.

We examined treatment patterns using ANCOVAs that controlled for all of the independent variables described above. To examine factors which affect emergency room visits among individuals diagnosed with ADHD, we conducted two multivariate analyses. Specifically, we used a logistic regression to examine the probability of being hospitalized during the twelve-month post-period, while we used a negative binomial regression for all patients to estimate the hospital length of stay over the post-period. As with the ANCOVA analyses, these multivariate regressions controlled for demographic characteristics, patient general health status, comorbid diagnoses, and the use of alternative ADHD medications. We considered findings of a p-value less than or equal to 0.05 to indicate statistical significance. We conducted all analyses using SAS Version 8.1 [25].

## Results

Table 1 presents the characteristics of the 5,939 individuals included in the analysis. Examining all individuals, the average age was approximately 15 (range 6–65), with the majority of individuals being 18 years of age or younger. The population consisted of more males than females (23% females), and the majority of individuals resided in the East (74%). The most common diagnoses were accident or injury (7%) and oppositional disorder (4%). The majority of individuals in the sample initiated therapy on OROS MPH rather than TID MPH (81% OROS MPH).

**Table 1:** Descriptive Statistics

**Variable**	**Entire Sample**	**OROS MPH**	**TID MPH**	**p Value**
**Demographics**				
Mean Age	14.81 (9.55)	14.37 (8.73)	16.62 (12.42)	< 0.0001
Female	23.49%	23.64%	22.88%	0.5849
Region				
East	73.60%	75.51%	65.58%	< 0.0001
South	3.00%	2.95%	3.21%	0.6426
North Central	1.38%	1.42%	1.21%	0.5869
West	7.27%	5.02%	16.64%	< 0.0001
HMO Insurance	35.65%	35.72%	35.37%	0.8185
**General Health Status**				
Prior number of diagnoses	3.34 (2.91)	3.44 (2.87)	2.96 (3.09)	< 0.0001
**Diagnoses**				
Anxiety	0.96%	0.92%	1.13%	0.5174
Depression	1.20%	1.13%	1.47%	0.3336
Oppositional disorder	4.45%	4.56%	3.99%	0.3992
Drug/Alcohol abuse	1.63%	1.65%	1.56%	0.8263
Accident/Injury	7.32%	7.42%	6.93%	0.5690
**Medication**				
Concerta	80.57%	100%	0%	NA
				
				
**Sample Size**	5,939	4,785	1,154	

Table 1 also presents the characteristics of the individuals who initiated therapy on OROS MPH compared to those individuals who initiated therapy on TID MPH. The OROS MPH cohort was significantly younger (mean age 14 vs. 17; p < 0.0001), had more individuals residing in the East (76% vs. 66%; p < 0.0001) and fewer individuals residing in the West (5% vs. 17%; p < 0.0001). In addition, the OROS MPH group had a significantly higher total number of diagnoses in the pre-period of analysis (3.44 vs. 2.96; p < 0.0001), although there were no significant differences between the two groups with regards to incidence of specific comorbid conditions associated with ADHD. There was also no difference between the OROS MPH and TID MPH groups with regards to gender (female; 24% vs. 23%, p = 0.5849).

Table 2 illustrates differences in treatment patterns in the OROS MPH and TID MPH cohorts. The mean length of therapy for the OROS MPH cohort was 199 days, compared to 107 days for the TID MPH cohort (p < 0.0001). Compared to individuals who received TID MPH, more individuals who initiated treatment with OROS MPH had either a 15-day gap in ADHD therapy (85% vs. 97%, p < 0.0001) or a 30 day gap in ADHD therapy (77% vs. 95%, p < 0.0001). In addition, the OROS MPH cohort was significantly fewer individuals switch to TID MPH than vice versa (1% vs. 33%, p < 0.0001) and significantly fewer individuals switch to any other ADHD medication (27% vs. 68%, p < 0.0001). There were significantly more individuals who received OROS MPH found to be adherent at either the 90%, 80% or 75% level (24% vs. 5%, p < 0.0001; 29% vs. 7%, p < 0.0001; 30% vs. 7%, p < 0.0001, respectively). As a test of the robustness of these results, we also examined adherence levels for the subset of individuals (N = 3,832) who did not switch ADHD medication over the course of the post-period. While the general adherence rates were higher in this subset of individuals, the general findings were consistent. Specifically, the OROS MPH group was more adherent at the 90%, 80% or 75% level (31% vs. 16%, p < 0.0001; 39% vs. 21%, p < 0.0001; 40% vs. 21%, p < 0.0001, respectively).

**Table 2:** Treatment Patterns – ANCOVA Analyses

**Variable**	**Mean OROS MPH**	**Mean TID MPH**	**Difference**	**p Value**	**95% Confidence Interval**
15 day Gap in ITT medication	0.85	0.97	0.11	<0.0001	0.0945 – 0.1366
30 day Gap in ITT medication	0.77	0.95	0.17	< 0.0001	0.1510 – 0.2022
Switch to another ADHD med	0.27	0.68	0.41	< 0.0001	0.3802 – 0.4394
Switch to other ITT med	0.01	0.33	0.31	< 0.0001	0.2962 – 0.3271
Days on ITT medication	199.09	107.73	-91.36	< 0.0001	-99.94 – -82.78
90% Compliant	0.24	0.05	-0.1818	< 0.0001	-0.2076 – -0.1560
80% Compliant	0.29	0.07	-0.2227	< 0.0001	-0.2503 – -0.1949
75% Compliant	0.30	0.05	-0.2274	< 0.0001	-0.2554 – -0.1995

ANCOVA results controlling for demographics, general health status, and diagnoses.

Table 3 examines the factors that affect the probability being hospitalized and the hospital length of stay for all individuals diagnosed with ADHD. The logistic regression captures how each of the independent variables affects the probability of being hospitalized, while the negative binomial regression of the coefficients for each of the respective independent variables measures how that variable affects the hospitalization length of stay. Given that these regressions allow for hospitalizations for any reason, we also examined the most frequent diagnoses associated with hospitalization. Such diagnoses included hyperkinetic syndrome of childhood (ICD-9 of 314.xx), disturbance of emotions specific to childhood and adolescence (ICD-9 of 313.xx), affective psychoses (ICD-9 of 296.xx), and depressive disorders, NOS (ICD-9 of 311.xx).

**Table 3:** Hospitalization – Multivariate Regression Analyses

**Variable**	**Dependent Variable – Hospitalization Logistic Regression**			**Dependent Variable – Hospital Length of Stay Negative Binomial Regression**		
	
	**Odds Ratio**	**95% Confidence Interval**	**p Value**	**Point Estimate**	**95% Confidence Interval**	**p Value**
**Demographics**						
Age	1.017	1.002 – 1.033	0.0234	-0.024	-0.040 – -0.089	0.0019
Female	0.953	0.634 – 1.432	0.8162	0.325	-0.099 – 0.0750	0.1333
East	0.724	0.476 – 1.099	0.1294	-0.795	-1.289 – -0.201	0.0016
South	1.395	0.573 – 3.397	0.4635	-1.364	-0.236 – 0.368	0.0073
North Central	0.401	0.050 – 3.183	0.3871	-1.807	-0.412 – 0.506	0.1256
HMO Insurance	0.976	0.667 – 1.428	0.8995	-0.332	-0.752 – 0.088	0.1213
**General Health Status**						
Prior number of diagnoses	1.086	1.035 – 1.138	0.0007	0.001	-0.044 – 0.047	0.9507
**Diagnoses**						
Anxiety	0.683	0.146 – 3.192	0.6380	0.527	-0.910 – 1.963	.4722
Depression	1.937	0.684 – 5.485	0.2132	-0.369	-1.395 – 0.656	.4804
Oppositional disorder	1.324	0.658 – 2.666	0.4318	1.155	0.432 – 1.878	.0017
Drug/Alcohol Abuse	9.332	5.328 – 16.344	< 0.0001	0.223	-0.287 – 0.732	.3913
Accident/Injury	2.340	1.446 – 3.735	0.6380	-0.619	-1.093 – 0.145	.0104
**Medication**						
OROS MPH	0.668	0.450 – 0.992	0.0454	-0.692	-0.157 – -0.228	.0035

As Table 3 illustrates, general health status and specific patient diagnoses also affected the probability of being hospitalized. As expected, individuals who were more seriously ill were significantly more likely to be hospitalized, with each increase in the number of diagnoses an individual had during the pre-period resulting in a 8% increase in the probability being hospitalized (p = 0.0007). Individuals diagnosed with drug or alcohol abuse were significantly more likely to be hospitalized (p < 0.0001), while individuals diagnosed as having an accident or injury were not less likely to be hospitalized but did have a significantly shorter length of stay (0.62 fewer days; p = 0.0104) in the one year post initiation on MPH medication. While a comorbid diagnosis of anxiety or depression was not found to have any impact on either the probability of being hospitalized or hospital length of stay, a diagnosis of oppositional disorder was associated with a significantly longer hospital length of stay, with individuals diagnosed with oppositional disorder staying, on average, 1.2 more days in the hospital (p = 0.0017).

Table 3 also illustrates that the formulation of MPH affects hospitalizations. Individuals who received OROS MPH were 33% less likely to be hospitalized compared to individuals who received TID MPH (OR = 0.67; p = 0.0454). The negative binomial regression results are largely consistent with this finding, with individuals who received OROS MPH having 0.69 fewer days hospitalized than individuals who received TID MPH (p = 0.0035).

To further test the robustness of the results, we re-examined each of the research questions omitting the criteria that individuals were required to have a diagnosis of ADHD. In addition, we performed the results with a 10% trim of the data in order to minimize the potential impact of outliers. In both cases, results are consistent with the findings reported.

## Discussion

These analyses provide evidence of several significant differences in both treatment patterns and outcomes between patients initiating treatment with OROS MPH and similar patients initiating treatment with TID MPH. The results of the multivariate regression analyses demonstrate that ADHD patients treated with OROS MPH are not only more likely to experience longer treatment periods than patients treated with TID MPH, but are significantly less likely to experience a gap in switch in therapy. Moreover, while examining compliance, defined as the number of days of medication an individual received over a one year period, those patients treated with OROS MPH are significantly more compliant to therapy than their counterparts treated with TID MPH. In addition, treatment initiation with OROS MPH is associated with significantly fewer hospitalizations as well as hospital stays of significantly shorter duration than treatment initiation with TID MPH, even after controlling for demographic variables, general health status, and co-morbid diagnoses.

The unadjusted differences between the OROS and TID groups revealed that individuals who received OROS had significantly more prior diagnoses than individuals in the TID cohort. It may be that individuals who are in poorer general health may be taking medications for other diagnoses and, as such, the OROS once-daily dosing may be particular appealing. However, it should be noted that there were no differences between the OROS and TID groups with regards to common comorbidities associated with ADHD.

Treatment patterns are of particular interest when comparing pharmacological therapies for ADHD as successful management of this disorder is characterized by both adherence to and compliance with a medication regimen [26]. Noncompliance to ADHD medication is considered a result of treatment frequency, dosing schedules, and the chronic nature of therapy and may be exacerbated by social stigma associated with taking medications, concerns over long-term safety, and inadequate monitoring [27,28]. Additionally, disease-related factors such as co-morbid oppositional and defiant behavior, easy distractibility, and poor self-regulation may also compromise medication compliance [27].

The results of this analysis highlighted better compliance, longer treatment periods, and fewer switches in the cohort initiating treatment with OROS MPH when compared to the TID MPH cohort. These results are not surprising considering Concerta's once-a-day administration coupled with previous research that found a significantly higher degree of compliance in patients on a once-a-day regime of MPH when compared to TID dosing [29]. While any stimulant medication may be at risk for suboptimal compliance and adherence [20] , it is clear that once-a-day administration can have a positive impact on these treatment patterns.

While this analysis found significant differences between OROS MPH and TID MPH relating to treatment patterns, the results also illustrate significant differences between the Concerta treatment regime and TID MPH when considering patient outcomes, defined as hospitalizations and length of hospital stay.

Previous research has found hospitalization to be a critical patient outcome to measure when examining ADHD. Leibson et al (2001) found ADHD patients more likely to experience a hospitalization than similar patients without ADHD [10]. Furthermore, among hospitalized patients, those carrying an ADHD diagnosis have been shown to experience significantly longer hospital stays than patients without a pre-hospitalization ADHD diagnosis [31-33]. Taking this research a step further, the present analyses sought to explore this outcome in the context of different dosing regimes of MPH therapy. After controlling for all other variables, the regression results find patients initiating treatment with once-a-day Concerta significantly less likely to receive a hospital admission, and more likely to experience a significantly shorter hospital stay than patients initiating treatment on TID MPH.

The Concerta treatment regime's impact on hospitalization has important implications when considering the direct medical costs of ADHD. In a study examining the costs of ADHD, Swensen et al (2003) found the average annual per-person hospital inpatient expenditures for ADHD patients to be $388 (1998 US dollars) compared with $103 for non-ADHD patients. Moreover, the hospital inpatient costs represented 24.7% of the total direct costs associated with ADHD [34]. This research, along with the current results of fewer hospitalizations and shorter stays in patients treated with OROS MPH, extends support for the proposal that once-a-day MPH medication may be able to alleviate some of the additional service use and costs associated with a diagnosis of ADHD.

Variables controlled for in the regression analyses included not only demographic variables and prior number of diagnoses, but diagnoses co-morbid to ADHD. Prior investigations of patients with ADHD suggest that these individuals have a significantly higher lifetime prevalence of oppositional disorder, mood and anxiety disorders, and/or substance abuse disorders than controls and as a consequence, experience an increased probability of inflated economic and social cost, including higher health care utilization [35]. Supporting these suggestions were the results of the present logistic regression analysis examining the risk of hospitalization among ADHD patients. The results indicate that a co-morbid diagnosis of drug/alcohol abuse significantly increases the risk of hospitalization. Similarly, the negative binomial regression model reveals a significant association between the co-morbid diagnosis of oppositional disorder and extended duration of hospital stay. Nevertheless, despite evidence of the frequent presentation of anxiety and depression with ADHD, the current analysis fails to demonstrate that either anxiety or depression co-morbid to ADHD affects either the risk of hospitalization or the length of hospital stay.

Interpreting the findings of these analyses must be performed in the context the study design's limitations. First, the cohorts included in the analyses were comprised of individuals continuously insured or continuously insured and employed for at least one year and who received a diagnosis of ADHD as well as receipt of an ADHD medication. While the size of the data set offers a wide geographic distribution, care must be taken when generalizing the results to other populations. Second, identification of individuals with ADHD was restricted to the use of diagnostic codes. This may not be as precise as formal diagnostic assessment for the identification of ADHD patients. Furthermore, such an identification does not allow for an examination of severity of ADHD illness. For example, while it may be that patients receiving TID MPH have exhibited more difficult behavior than the OROS group, this analysis is unable to directly examine this issue. Third, the use of medical claims data prevents inclusion of potentially influential factors, such as ethnicity, into the analyses. Furthermore, the medical claims data used in this study does not allow for a direct examination of the potential impact of benefit design. Fourth, this analysis can not control for planned reductions in use of medications such as a stop in medication usage in the summer or a reduction in dosage from three-times-daily to twice-daily. Finally, medical claims data and employment records do not include patient assessments, thus precluding examination of quality of life, functioning, or clinical outcomes.

## Conclusion

Treatment patterns and patient outcomes among ADHD patients initiating treatment with OROS MPH or TID MPH were explored through this retrospective analysis of administrative claims. The results reveal that OROS MPH is significantly associated with longer treatment periods, fewer therapy switches, increased medication compliance, fewer hospitalizations, and shorter hospital stays when compared with patients receiving TID MPH. In sum, this study provides evidence that once-a-day administration of methylphenidate may offer improved compliance and adherence imperative to successful ADHD management as well as reduced utilization of hospital services.

## Competing interests

Funding for this study was provided by McNeil Consumer and Specialty Pharmaceuticals.

## Authors' contributions

JK and ML conceptualized and designed the study. ML had primary responsibility for analysis of and interpretation of data, as well as drafting the manuscript. JK provided critical revisions of the manscript.
